# In Vivo Disintegration of Four Different Luting Agents

**DOI:** 10.1155/2012/831508

**Published:** 2011-10-05

**Authors:** Deniz Gemalmaz, Cornelis H. Pameijer, Mark Latta, Ferah Kuybulu, Toros Alcan

**Affiliations:** ^1^Department of Prosthetic Dentistry, School of Dentistry, Marmara University, 34726 Istanbul, Turkey; ^2^Department of Reconstructive Sciences, University of Connecticut, Farmington, CT 06030, USA; ^3^School of Dentistry, Creighton University, Omaha, NE 68178, USA; ^4^Department of Orthodontics, School of Dentistry, Marmara University, 34365 Istanbul, Turkey

## Abstract

The purpose of this study was to evaluate the disintegration of luting agents. An intraoral sample holder was made having four holes of 1.4 mm diameter and 2 mm depth. The holder was soldered onto the buccal surface of an orthodontic band, which was cemented to the first upper molar in 12 patients, average age 26 years. The holes were filled with a zinc phosphate (Phosphate Kulzer), a glass ionomer (Ketac Cem), a resin-modified-glass ionomer (Fuji Plus), and a resin cement (Calibra). Impressions were made at baseline, and 6, 12, and 18 months from which epoxy replicas were made, which were scanned with an optical scanner. Total volume loss was calculated. The rank order of mean volume loss was as follows: Phosphate cement > Ketac Cem = Fuji Plus = Calibra. Cement type and time had statistically significant effects on volume loss of cements (*P* < 0.001). Under in vivo conditions, zinc phosphate cement disintegrated the most, whereas no significant difference was observed for glass ionomer and resin-based cements. As intraoral conditions are considerably less aggressive than experimental laboratory conditions, the erosion behavior of glass ionomer cement was found to be similar to the resin-based cements in contradiction to previous laboratory results.

## 1. Introduction

Solubility and disintegration of luting cements are important factors that determine the clinical longevity of crowns, bridges, posts, and so forth. Numerous articles have appeared in the literature evaluating these properties [1–11]. Among the factors that complicate direct comparisons when employing in vitro test are differences in the chemical composition of the various cements and hence different mechanisms of breakdown. The most common laboratory test on solubility and disintegration can be found in the ADA specification no. 8 in which a cement disc is immersed in distilled water for 24 h, after which the solute is gravimetrically determined [12]. The ADA test does not give an indication of the fully hardened cement, either in water or in oral fluids [13]. Many investigators have reservations about this test and have used different methods [6, 14–18]. Some authors designed a laboratory test that better correlated with in vivo disintegration results or varied the test circumstances by using longer periods and/or different pH levels [6, 14, 18]. In addition, artificial and natural saliva [15] were used, and passive or agitation conditions were introduced [6, 7, 9, 14, 15]. Other investigators [16–18] concentrated on the erosion factor and directed jets of media onto the surfaces of specimens. The results demonstrated that the combined solubility/erosion in a liquid flow results in different relative deterioration rates compared to a nonflowing medium [18].

Attempts to correlate in vitro solubility with the rate of degradation in the oral cavity has been limited. One of the early reports dates back to 1969 [2], in which cast removable partial dentures were used. The cements were placed in relatively large cavities located on the lingual surface of the denture and worn for 30 days by 8 patients. The appliances were weighed, and the loss of cement was calculated. Cements that were tested were zinc phosphate, silicate, and zinc oxide eugenol cements, and the changes reported were subsequently 5–30 mg·cm^−2^, 0 mg·cm^−2^, and 20–100 mg·cm^−2^ [2].

Other studies that focused on in vivo disintegration of luting cements have used various designs such as placing cements into molds of different sizes either in removable or fixed partial dentures.  Table 1 is a summary of the current literature evaluating in vivo disintegration of luting cements. A comparison of the results from relevant literature is challenging due to the difference in tested cements, evaluation times and variation in exposed surface areas. However, most studies have focused on in vivo disintegration of acid-base reaction cements demonstrating that from lowest to highest disintegration the sequence was glass ionomer cement < zinc phosphate < polycarboxylate cement [5, 7, 10, 19].

The introduction of new adhesive techniques and materials for use in restorative dentistry also led the development of new dental cements that are resin based. The ability to adhere to multiple substrates, high strength, and insolubility in the oral environment are major advantages of the resin-based luting agents. However, the number of clinical studies regarding the disintegration of these new luting agents is limited. Roulet and Wälti [8] used a “drawer” made from type II gold in the pontic of thirteen sanitary-type fixed partial dentures. They showed the behavior of a composite resin and a glass-ionomer cement in the oral environment in which composite resin exhibited improved resistance to solubility in comparison to glass ionomer material during 28 months of in vivo conditions.

The aim of this study was to evaluate the in vivo disintegration of resin-based luting cements (resin-modified-glass ionomer and resin cement) in comparison to zinc phosphate and a glass ionomer cement over a period of 18 months.

## 2. Subjects and Methods

**Figure 1:**
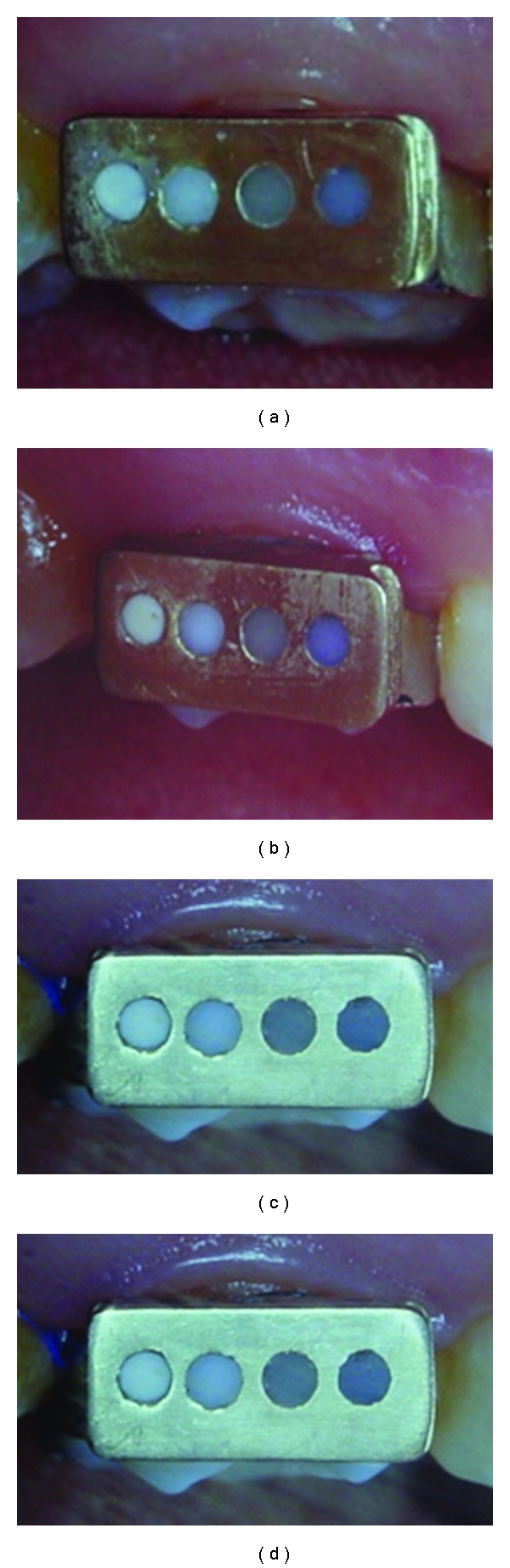
Cement holder (a) at baseline; (b) after 6 months; (c) after 12 months; and (d) after 18 months. Cements were placed in uniform cylindrical openings. In sequence from left to right are zinc phosphate, glass ionomer, resin-modified glass ionomer, and resin cement.

Twelve intraoral sample holders (9 mm high, 5 mm wide, and 2.5 mm deep) designed as a receptacle for the test materials were fabricated in gold (Biopontostar alloy, Bego, Bremen, Germany). Each holder had four holes, 1.4 mm in diameter and 2 mm deep, prepared perpendicular to the surface. Each holder was soldered to the buccal surface of an orthodontic band which was suitable for placement on the upper first molar. Each opening contained a different cement: (1) a zinc phosphate cement (Phosphate), (2) a glass ionomer cement (Ketac Cem), (3) a resin modified glass ionomer cement (Fuji Plus), and (4) a resin cement (Calibra) (see Figure 1).

The luting agents were mixed at room temperature on a glass slab by the same person, using a stainless steel spatula with a stiff blade. The powder/liquid (P/L) ratios were in accordance with the manufacturers' recommendations and are presented in Table 2. Powder and liquid were weighed using an electronic digital microbalance (Scaltec, Hamburg, Germany) with a measuring accuracy of 0.1 mg. To reduce the possibility of voids, a spiral lentulo was used for insertion of the cement into the openings. After a slight overfill was established, a myler strip covered the cement and was pressed down with a glass plate, which was retained with a spring clamp. During setting, the sample holder was placed in an incubator with a relative humidity of 100% at 37°C for 10 minutes. The resin-based cement was inserted into the openings as described above followed by light curing for 40 s (Eliza Light 500, Apoza Enterprise Co., Ltd., Taipei Hsien, Taiwan). After curing or setting, the surface of the sample holder containing the cements was finished to a uniform flat surface on wet 600-grit paper (English Abrasives and Chemicals Ltd., Manchester, England). 

The study design had been approved by the Ethics Board of Marmara University, Istanbul, Turkey. The objectives of the study were explained to the patients and informed consent was obtained. Criteria for inclusion included (1) ≥18 years of age, (2) upper first molar free of caries, and (3) good oral hygiene. Patients were also questioned on their dietary intakes in order to exclude individuals whi frequently consumed low pH foods or had bulemia. 

Prior to cementation of the appliance an impression was made of the surface of the cement holder using a vinyl polysiloxane impression material (Panasil contact plus, Kettenbach Dental, Germany). The replica made thereof established the baseline. The appliance was then cemented to an upper first molar of each patient. Routine oral hygiene instructions were reviewed and a standard soft brush (Colgate Total Soft, Colgate-Palmolive Co.) and a microabrasive toothpaste (Colgate Mint Stripe Gel, Colgate-Palmolive Co.) were supplied. The patients were recalled at 6, 12 and 18 months. At each recall visit, an impression of the cement holder was made intraorally using the previously described impression material. The negative impression was poured with an epoxy resin resulting in a replica of the cements in the holder. A Proscan 2000 A (Scantron Inc.) noncontact optical scanner was used for analyzing the epoxy replicas. A S5/03 sensor was used with a resolution of 0.01 micron and differences between the baseline replicas and the 6, 12, and 18 month samples represented total volume loss. A 2-way analysis of variance and Tukey multiple range tests were performed to distinguish statistically significant differences between the groups (*P*  <  .001).

## 3. Results

The mean and standard deviations of the volume loss of luting agents are shown in Table 3. The lowest cement loss was recorded for Calibra after 6 months (<0.005 mm^3^), whereas Phosphate cement after 18 months recorded the greatest loss (0.31 mm^3^). Of all luting agents, Phosphate cement showed the highest mean loss of substance at all observation times. Increasing the observation time resulted in a marked increase in loss from the surface of Phosphate cement. 

ANOVA showed that cement type, time, and their interactions all had a statistically significant effect on volume loss (*P*  <  .001). Tukey multiple range test revealed that the volume loss of zinc phosphate cement was statistically significantly greater than Ketac Cem, whereas no significant differences were observed between Ketac Cem, Fuji Plus, and Calibra.

## 4. Discussion

The disintegration of a luting agent is an important factor affecting the long-term durability of a restoration. Mechanical wear due to brushing abrasion and chewing forces, leaching due to chemical erosion, and fatigue of the small amount of luting material at the margins as a result of mechanical loading, are the factors that complicate the disintegration mechanism of a luting agent. In this respect, several in vitro studies [6, 7, 9, 14, 15] have appeared in the literature reporting on the solubility and disintegration of luting agents. There have been attempts at simulating the complexity of the oral environment in these in vitro tests. Despite in vitro studies supplied knowledge on mechanical properties of the materials used, no in vitro method can totally subject materials to in vivo conditions, since they can not simulate the pH and temperature changes of the oral cavity. Thus, the correlation between the results of in vitro studies and clinical studies has to be questioned.

 There are few clinical studies [5, 7, 10, 19, 20] of adequate duration to establish correlation with in vitro testing. In addition, most of the existing in vivo disintegration studies [5, 7, 10, 19, 20] have focused on acid-base reaction cements, and only one clinical study [8] investigating a resin-based cement has been reported. Roulet and Wälti [8] designed a special “drawer”, in the pontic of thirteen sanitary-type fixed partial dentures, to show the behavior of a composite resin and a glass-ionomer cement in the oral environment by excluding the effect of mechanical wear caused by opposing teeth, food, or toothbrushing. A glass-ionomer cement and composite resin were inserted in cavities of the drawer and material loss was measured with a three-coordinate measuring machine after 2, 9, 16, and 28 months. They showed that composite resin exhibited decreased solubility in comparison to glass ionomer material after 28 months evaluation time. 

 In the present study, disintegration of resin-based luting agents (a resin-modified-glass ionomer and a resin cement) were evaluated in comparison to zinc phosphate and glass ionomer cements over a period of 18 months. To more reliably correlate in vivo data with laboratory conditions, a previous study [11] evaluated the erosion of the same cements by means of immersion in 0.1 M aqueous sodium lactate/lactic acid buffer (pH = 2.74 and 4.0) over a period of 28 days. The results of the current study confirmed that zinc phosphate cement showed higher disintegration in comparison to glass ionomer and resin-based cements. In the in vitro study [11], studying identical cements, zinc phosphate cement also exhibited higher solubility than glass ionomer cement and resin cements in pH values of either 2.74 and 4.0. Various in vitro and in vivo studies have shown that zinc phosphate cement has higher solubility compared to glass ionomer cement [5, 7, 10, 19, 20]. The deterioration of phosphate cement is due to the loss of zinc from the matrix of phosphate cement, whereas the composition of glass ionomer cement remained nearly constant due to the setting reaction between the fluoroaluminosilicate glass and polyacrylic acid [18, 21].

 The glass ionomer cement (Ketac Cem) tested in this study did not exhibit significantly higher disintegration compared to the resin-based luting cements which is in contrast to the findings of in vitro studies [11, 22]. Most in vitro solubility experiments were performed at one or two pH values, mainly at pH 2.7 or pH 4 or higher. Increased solubility at low pH compared to neutral conditions was a common finding for water-based cements, which occurred linear with time [18, 22]. It appears that in vitro experimental designs are static solubility tests, as they use a constant low pH lactate acid. As laboratory conditions are more aggressive than clinical conditions, the aggressive acidic conditions generated greater loss of glass ionomer cement in comparison to resin-based luting cements.

 In a recent study, Meşe et al. [21] used a modified ISO 4049 test to evaluate sorption and solubility of 8 resin-based luting agents in two different solutions: 50% ethanol and water. They demonstrated that in water and in an ethanol/water solution, resin-modified glass ionomer cements exhibited higher sorption and solubility as compared to resin-based luting cements. Resin-modified glass ionomer cement, GC Fuji Plus, also exhibited significantly higher solubility when compared to resin-based luting agents in both water and ethanol/water. The authors contributed the significantly higher solubility of Fuji Plus to the likelihood of unpolymerized free monomers being leached out in an aqueous environment. In the present study, no significant differences were observed between glass ionomer (Ketac Cem), resin-modified glass ionomer (Fuji Plus), and resin cement (Calibra). It is postulated that the less aggressive conditions of the oral environment resulted in less erosion of the glass ionomer, resin-modified glass ionomer and resin cements. Intermittent exposure to acidic solutions, salivary flow, and buffering capacity is one of the factors that contributed to less disintegration of the test cements under in vivo conditions. Salivary pH and buffering capacity are believed to be the sole factors that can effect cement solubility. However, Pluim [18] showed in an in vivo study that there was no correlation between salivary pH and buffering capacity and cement solubility. They concluded that cement erosion was due to bacterial and dietary acids and not to dissolution by saliva. Thus, the differences between the cement loss values of individuals can be attributed to the differences between their dietary intakes.

It would be an error to predict in vivo disintegration of luting materials from this in vivo experiment, as the exposed cement surface of 1.4 mm in diameter was a factor many times larger than a clinically acceptable margin of 40 *μ*m. Thus, the level of erosion should be considered within the context of exposed surface area. The luting agents used in fixed prosthesis with clinically acceptable marginal adaptation will be subjected to less tooth brush abrasion and wear from chewing and will most probably show lower disintegration rates.

## 5. Conclusions

The results of this in vivo study support the conclusions that:

1. zinc-phosphate cement showed greater disintegration than glass ionomer and resin-based cements whereas the mean disintegration values of glass-ionomer and resin-based cements were not significantly different,
2. as intraoral conditions are considerably less aggressive than experimental laboratory conditions the erosion behaviour of glass ionomer cement was found to be similar with the resin-based cements in contradiction to previous laboratory results.

## Figures and Tables

**Table 1:** In vivo disintegration studies on luting cements.

Author, year	Exposed cement surface area	Duration	Cement	Disintegration
Norman et al. [2], 1969	Large cavity extending from lower premolar to molar region	30 days	ZnP S ZOE/EBA	5–30 mg/cm^2^ 0 mg/cm^2^ 20–100 mg/cm^2^

Richter and Ueno [3], 1975	3 mm diameter	12 months	SP ZnP ZOE/EBA PC	SP<ZP<PC *≃* ZOE/EBA

Osborne et al. [4], 1978	0.82 mm diameter	6 months	SP ZnP ZOE/EBA PC	SP<ZP<PC< ZOE/EB

Mitchem and Gronas [5], 1978	2 mm diameter	6 months	GIC SP ZnP PC	200 *μ*m 350 *μ*m 600 *μ*m 930 *μ*m

Sidler and Strub [20], 1983	0.8 mm diameter	14 months	GIC ZnP	40–100 *μ*m 500 *μ*m

Roulet and Wälti [8], 1984	1.5 mm diameter	28 months	GIC R (Adaptic) R (Adaptic + Soflex disc)	0,041 mm 0,0086 mm 0,01 mm

Phillips et al. [19], 1987	0.8 mm diameter	6–12 months	GIC SP PC (High ratio of powder to liquid) ZnP	GIC<SP<PC< ZP

Pluim et al. [7], 1984	1.3 mm diameter	6 months	GIC ZnP PC	0,5–1 *μ*m/week 20–22 *μ*m/week 18–30 *μ*m/week

Hersek and Canay [10], 1996	5 mm diameter	8 months	PC ZnP GIC	GIC<ZP<PC

ZOE/EBA, zinc oxide eugenol reinforced with ethoxybenzoic acid; S, silicate cement; SP, silicophosphate cement; PC, polycarboxylate cement; ZnP, zinc phosphate cement; GIC, glass ionomer cement; R, composite resin.

**Table 2:** Luting cements used in study.

Product name	Producer	Batch no.	Type	P/L Ratio (g)
Phosphate cement	Heraeus Kulzer, Werheim, Germany	P: 1650437 L: 1750438	Zinc Phosphate	1.2/0.88
Ketac Cem	3M ESPE, Germany	P: 138783 L: 129918	Glass Ionomer	3.8/1
Fuji Plus Capsule	GC Corporation, Tokyo, Japan	0303262	Resin-modified glass ionomer	2/1
Calibra	Dentsply Caulk, USA	Base: 0208141 Catalyst: 030108	Resin	(Base/Catalyst) 1

P, powder; L, liquid.

**Table 3:** Mean volume loss (mm^3^) observed for the tested cements as a function of time.

	6 months	12 months	18 months
Zinc Phosphate	0,13 ± 0,05	0,24 ± 0,05	0,31 ± 0,09
Ketac Cem	**0,03 ± 0,03**	**0,05 ± 0,03**	**0,08 ± 0,06**
Fuji Plus	**0,02 ± 0,02**	**0,05 ± 0,04**	**0,08 ± 0,07**
Calibra	**<0,005**	**0,01 ± 0,01**	**0,02 ± 0,02**

Means expressed in bold are not statistically significant.
